# Passive Resistor Temperature Compensation for a High-Temperature Piezoresistive Pressure Sensor

**DOI:** 10.3390/s16071142

**Published:** 2016-07-22

**Authors:** Zong Yao, Ting Liang, Pinggang Jia, Yingping Hong, Lei Qi, Cheng Lei, Bin Zhang, Wangwang Li, Diya Zhang, Jijun Xiong

**Affiliations:** 1 National Key Laboratory for Electronic Measurement Technology, North University of China, Taiyuan 030051, China; yaozong126@sina.com (Z.Y.); pgjia@cqu.edu.cn (P.J.); hongyingping_2014@163.com (Y.H.); qilei19850224@163.com (L.Q.); leichengnuc@163.com (C.L.); zb0003@126.com (B.Z.); 18434365707@163.com (W.L.); 18234157832@163.com (D.Z.); 2 Key Laboratory for Instrumentation Science & Dynamic Measurement, North University of China, Ministry of Education, Taiyuan 030051, China

**Keywords:** high-temperature piezoresistive pressure sensor, passive resistor, temperature compensation

## Abstract

The main limitation of high-temperature piezoresistive pressure sensors is the variation of output voltage with operating temperature, which seriously reduces their measurement accuracy. This paper presents a passive resistor temperature compensation technique whose parameters are calculated using differential equations. Unlike traditional experiential arithmetic, the differential equations are independent of the parameter deviation among the piezoresistors of the microelectromechanical pressure sensor and the residual stress caused by the fabrication process or a mismatch in the thermal expansion coefficients. The differential equations are solved using calibration data from uncompensated high-temperature piezoresistive pressure sensors. Tests conducted on the calibrated equipment at various temperatures and pressures show that the passive resistor temperature compensation produces a remarkable effect. Additionally, a high-temperature signal-conditioning circuit is used to improve the output sensitivity of the sensor, which can be reduced by the temperature compensation. Compared to traditional experiential arithmetic, the proposed passive resistor temperature compensation technique exhibits less temperature drift and is expected to be highly applicable for pressure measurements in harsh environments with large temperature variations.

## 1. Introduction

In recent years, universal high-temperature piezoresistive pressure sensors have been extensively applied in the fields of petrochemicals, energy and electric power, and aerospace. Thus, improving the measurement accuracy is likely to play an increasingly important role in various industries, especially those that encounter high-temperature environments [1]. Among the current applications, sensors are expected to deliver consistent performance and accuracy under temperature increases of up to 220 °C. In such situations, however, a continuous drift in the sensor’s output voltage is inevitable, because the piezoresistance increases with temperature, whereas the piezoresistive coefficient decreases [2], which adversely affects testing precision. Hence, it is critical to achieve effective temperature compensation for high-temperature piezoresistive pressure sensors.

The main measuring principle used by high-temperature piezoresistive pressure sensors is the Wheatstone bridge. The output voltage of a Wheatstone bridge must be compensated in terms of the offset voltage, the temperature coefficient of the offset, and the temperature coefficient of the sensitivity. Current bridge temperature compensation methods can be divided into hardware, software, or hybrid techniques. Hardware methods typically use an extra thermistor, a low-temperature coefficient resistor network, a diode, a triode, an adjustable gain operational amplifier, and so on [3]. The software methods are based on data processing according to an inverse function algorithm or artificial neural networks [4,5]. Hybrid techniques combine the hardware and software approaches to obtain higher compensation accuracy. Compared with other techniques, hardware compensation is simpler, more efficient, more economical, and easier to implement in industrial production and manufacturing; hence, hardware compensation is widely used.

**Figure 1:**
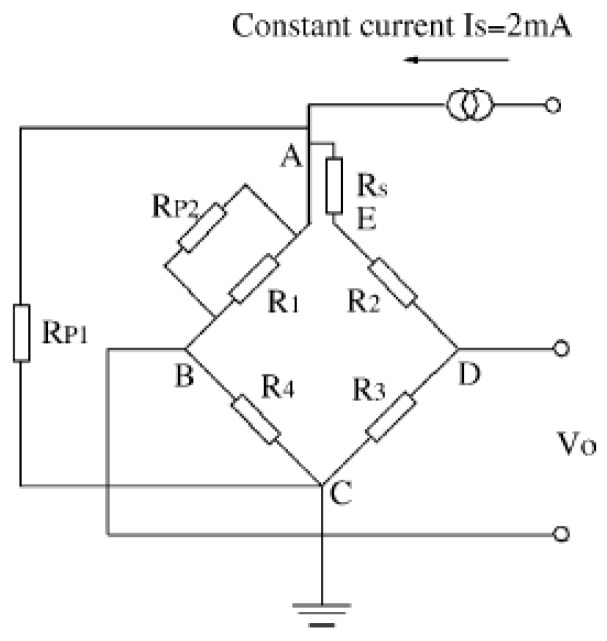
Typical compensation circuit in a low-temperature coefficient resistor network.

In this study, we describe a passive resistor temperature compensation technique that uses a low-temperature coefficient resistance network. Unlike other hardware methods, which require the compensation circuit to be in the same temperature field as the bridge arm piezoresistor, our passive resistor temperature compensation circuit does not need to be at the working temperature of the bridge arm piezoresistor, allowing more flexibility and convenience in packing. Moreover, the compensation accuracy of the proposed method is independent of the temperature difference between the compensation circuit and the bridge arm piezoresistor. A typical circuit diagram of a low-temperature coefficient resistor network model is shown in Figure 1. 

The traditional theoretical compensation formulas for the offset voltage and temperature coefficient of the offset are given in Equations (1) and (2), respectively; the compensation formula for the temperature coefficient of the sensitivity is given in Equation (3).

(1) $$R_{S}={\text{KR}}_{B}$$

(2) $$R_{P2}=\frac{\left(1+K\right)\left(\sqrt{1+K}+1\right)}{K}R_{B}\approx \frac{2R_{B}}{K}，K=\frac{4V_{\text{OS}}}{V_{B}}$$

(3) $$R_{P1}=-\frac{\alpha }{\alpha {+\text{TCR}}_{B}}R_{B}$$

where R_B_ is the bridge arm resistance, TCR_B_ is the temperature coefficient of R_B_, V_OS_ is the bridge zero output voltage, and α is the temperature coefficient of the output voltage sensitivity [6].

These equations assume that the four bridge arm resistances and their temperature coefficients have almost the same initial values, and neglect the influence of residual stress caused by the fabrication process. Otherwise, the compensation applied to the temperature coefficient of the offset and the sensitivity would be invalid [7,8]. In contrast, the passive resistor temperature compensation algorithm proposed in the next section uses actual sensor measurement data, and is independent of residual stress and deviations in the bridge parameters. This results in high compensation precision and extensive applicability.

## 2. Principles of Passive Resistor Temperature Compensation

Passive resistor temperature compensation is based on the assumption that passive resistance is temperature-independent. This means that the temperature coefficient of the passive resistor should be less than 10% of that of the bridge arm resistor. In this case, the temperature coefficient of the passive resistor can be ignored. As the temperature coefficient of a typical silicon piezoresistor is greater than 2000 ppm [9], the temperature coefficient of the passive resistor should be less than 200 ppm.

### 2.1. Compensation of Offset Voltage and Temperature Coefficient of Offset

The temperature of the bridge arm resistor R_t_ can be expressed as:

(4) $$R_{t}=R_{0}[1+\alpha_{R}(t-t_{0})]$$

The temperature coefficient expression α_R_ is given by:

(5) $$\alpha_{R}=\frac{R_{t}-R_{0}}{R_{0}}\times \frac{1}{t-t_{0}}$$

Compensation can be accomplished by correcting the temperature coefficient of the bridge arm resistor. This can be done using simple circuit techniques with passive shunt or series resistor elements with temperature-independent resistance.

**Figure 2:**
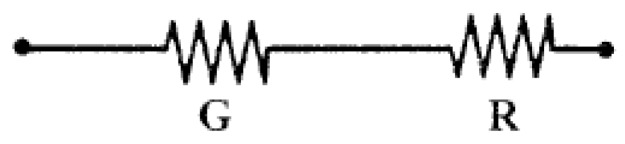
Serial connection for compensation of the bridge offset output voltage.

Figure 2 illustrates the serial placement of the resistor G, which has a near-zero temperature coefficient of resistance. The total temperature coefficient of G and R_t_ is α_R+G_, as shown in Equation (6). A comparison of Equations (5) and (6) shows that α_R+G_ is less than α_R_. This implies that the temperature coefficient of the bridge arm resistor with a series compensation resistor is less than that of the bridge arm resistor itself.

(6) $$\alpha_{R+G}=\frac{\left(R_{t}+G)-(R_{0}+G\right)}{R_{0}+G}\times \frac{1}{t-t_{0}}=\frac{R_{t}-R_{0}}{R_{0}+G}\times \frac{1}{t-t_{0}}$$

**Figure 3:**
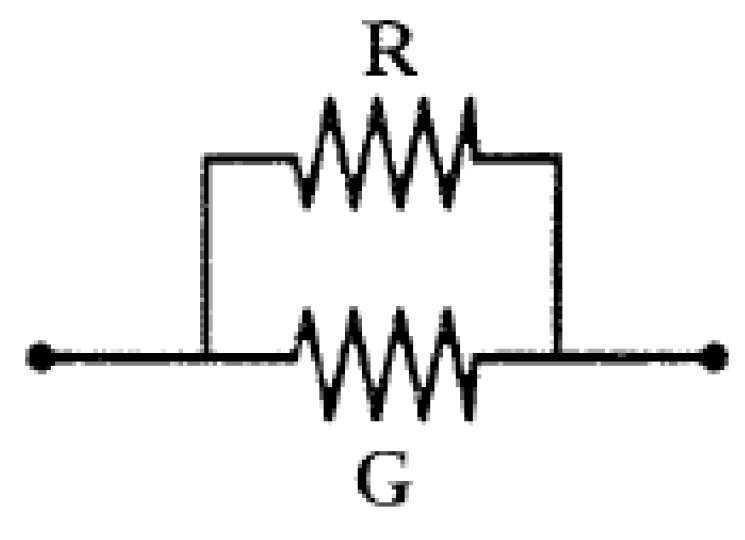
Parallel connection for compensation of the bridge offset output voltage.

Figure 3 shows the shunt placement of resistor G. The total temperature coefficient of resistor G and R_t_ is α_R‖G_, as shown in Equation (7). A comparison with Equation (5) shows that α_R‖G_ is less than α_R_, which means that the temperature coefficient of the bridge arm resistor with a shunt compensation resistor is also less than that of the bridge arm resistor itself.

(7) $$\alpha_{R\|G}=\frac{R_{t}\|G-R_{0}\|G}{R_{0}\|G}\times \frac{1}{t-t_{0}}=\frac{G}{R_{0}+G}\left(\frac{R_{t}-R_{0}}{R_{0}}\times \frac{1}{t-t_{0}}\right)=\frac{1}{\frac{R_{t}}{G}+1}\times \alpha_{B}$$

where the symbol ‘‖’ represents that the two resistors are connected in a parallel relationship.

From the above analysis, we can infer that increasing the serial compensation resistance or decreasing the shunt compensation resistance can reduce the temperature coefficient of the bridge arm resistor with a compensation resistor and adjust the offset. This is the principle of compensation for the offset voltage and the temperature coefficient of offset.

### 2.2. Compensation of Temperature Coefficient of Sensitivity

**Figure 4:**
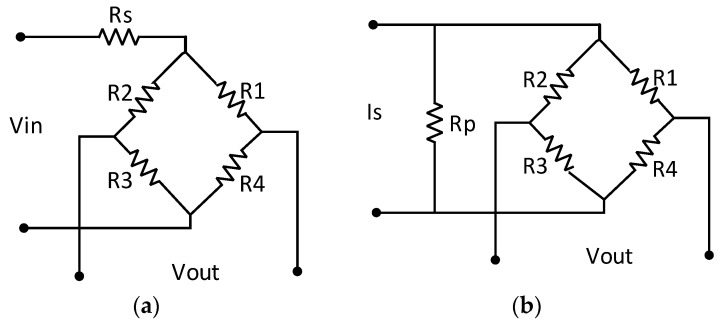
Compensation of the bridge sensitivity.

The temperature coefficient of sensitivity can be compensated using a serial temperature-independent resistor R_s_ under a constant voltage supply, as shown in Figure 4a, and a parallel temperature-independent resistor R_P_ under a constant current supply, as shown in Figure 4b.

For the example illustrated in Figure 4a, the output voltage of the bridge is expressed by Equation (8), where V_IN_ is the supply voltage, R_B_(T) is the equivalent bridge arm resistor, S(T) is the sensitivity, and P is the load pressure. As the temperature rises, R_B_(T) increases and S(T) decreases. Obviously, the temperature drift of the sensitivity can be compensated by selecting a suitable value of R_S_ according to Equation (8).

(8) $$V_{\text{OUT}}=V_{\text{IN}}（\frac{R_{B}（T）}{R_{B}（T）+R_{S}}）\times S(T)\times P$$

A simple qualitative explanation of how the compensation resistor R_S_ works is that, as the temperature rises, the resistance of the bridge R_B_(T) increases, whereas the resistance of R_S_ is almost unchanged with a near-zero temperature coefficient. This leads to an increase in the proportion of the supply voltage in the bridge and an increase in the bridge output voltage. The resistance of R_S_ is chosen such that the decrease in the sensitivity of the bridge as the temperature increases can be compensated by the increase in the bridge output voltage with temperature. A similar interpretation can be applied to the case of a constant current supply, as in Figure 4b. 

## 3. Model and Algorithm for Passive Resistor Temperature Compensation

**Figure 5:**
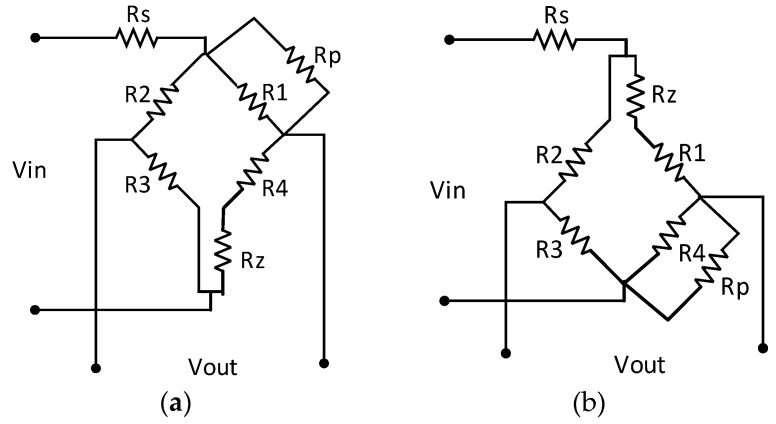
Passive resistor temperature compensation model with a constant voltage supply: **(a**) negative initial offset voltage; (**b**) positive initial offset voltage.

Our model for passive resistor temperature compensation is based on the principles described above. As a simple supply circuit is best suited to high-temperature applications, a constant voltage supply is usually adopted by high-temperature pressure sensors. The following describes the model and algorithm for passive resistor temperature compensation with a constant voltage supply. Figure 5 illustrates the model.

The sensor output voltage is:

(9) $$V_{\text{OUT}}\left(T,P\right)=V_{B}\left(T,P\right)\times \left[K_{+}\left(T,P\right)-K_{\_}\left(T,P\right)\right]$$

where K_+_ (T, P) and K_-_ (T, P) are the positive and negative voltage division factors, respectively, and V_B_(T, P) is the supply voltage of the bridge, which can be written as:

(10) $$V_{B}\left(T,P\right)=V_{\text{IN}}\times \frac{R_{B}\left(T,P\right)}{R_{B}\left(T,P\right)+R_{S}}$$

According to the initial offset voltage and sensor test data, we select the compensation model in Figure 5a or that in Figure 5b and the corresponding computational formula. The measurement parameters of the compensation model, which should be tested in advance, involve the four bridge arm resistances at the two compensation temperature thresholds of the high-temperature pressure sensor T0, T1 (T0 < T1) and two load pressures P0, P1 (P0 < P1). These values are presented in Table 1.

The compensation model in Figure 5a can be analyzed as follows:

Bridge resistance:

(11) $$R_{B}\left(T,P\right)=\left[R_{2}\left(T,P\right)+R_{3}\left(T,P\right)\right]\left[R_{Z}+R_{4}\left(T,P\right)+R_{1}\left(T,P\right)\|R_{P}\right]$$

Voltage division factors:

(12) $$K_{+}\left(T,P\right)=\frac{R_{Z}+R_{4}\left(T,P\right)}{R_{Z}+R_{4}\left(T,P\right)+R_{1}\left(T,P\right)\|R_{P}}$$

(13) $$K_{\_}\left(T,P\right)=\frac{R_{3}\left(T,P\right)}{R_{2}\left(T,P\right)+R_{3}\left(T,P\right)}$$

The output voltage expression of the model in Figure 5a can be described by substituting Equations (10)–(13) into Equation (9). The output voltage expression of the model in Figure 5b can be described in a similar way. This gives:

(14) $$\begin{array}{cc} V_{OUT}(T,P)=V_{IN} & \times \frac{\left[R_{2}\left(T,P\right)+R_{3}\left(T,P\right)]\|[R_{Z}+R_{4}\left(T,P\right)+R_{1}\left(T,P\right)\|R_{p}\right]}{[R_{2}\left(T,P\right)+R_{3}\left(T,P\right)]\|[R_{Z}+R_{4}\left(T,P\right)+R_{1}\left(T,P\right)\|R_{p}]+R_{S}}\\ & \times [\frac{R_{Z}R_{3}\left(T,P\right)}{R_{Z}R_{4}\left(T,P\right)+R_{1}\left(T,P\right)\|R_{p}}-\frac{R_{3}\left(T,P\right)}{R_{2}\left(T,P\right)+R_{3}\left(T,P\right)}] \end{array}$$

where R_Z_, R_P_, R_S_ are the model parameters to be determined, and R_i_(T, P) (i = 1, 2, 3, 4) are the known measurement parameter values in Table 1.

According to the demands of the bridge temperature compensation, the algorithm for passive resistor temperature compensation can be written as:

(15) $$\left\{ \begin{array}{c} T_{\text{OUT}}(T_{0},P_{0})=U_{0}\text{ Compensation of offset voltage }U_{0}\\ \frac{\partial V_{\text{OUT}}(V,P_{0})}{\partial T}=0\text{ Compensation of temperature coefficient of offset}\\ \frac{\partial V_{\text{OUT}}(V,P_{1})}{\partial T}=0\text{ Compensation of temperature coefficient of sensitivity} \end{array}\right.$$

It can be seen from these equations that compensating for the temperature coefficient of offset (sensitivity) requires the sensor output voltage under the initial (higher) load pressure P_0_ (P_1_) to be independent of the temperature. This means that the partial derivatives of V_OUT_ (T, P_0_) and V_OUT_ (T, P_1_) with respect to temperature T must remain equal to zero.

The values of R_Z_, R_P_, R_S_ in the passive resistor temperature compensation model can be determined from Equation (15) using computer software such as MATLAB [10].

## 4. Experiments and Data Processing

**Figure 6:**
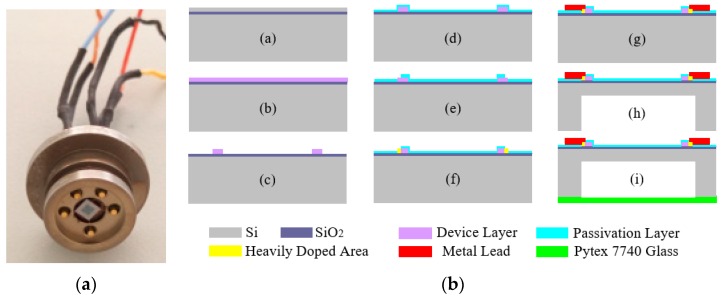
The developed high-temperature pressure sensor and fabrication process: (**a**) the high-temperature pressure sensor; (**b**) the MEMS fabrication process.

Figure 6a shows an uncompensated high-temperature pressure sensor based on silicon on insulation (SOI) material that can work for long periods within a temperature range of 220 °C. The standard microelectromechanical system (MEMS) processes shown in Figure 6b were used to fabricate this pressure-sensitive chip.

**Figure 7:**
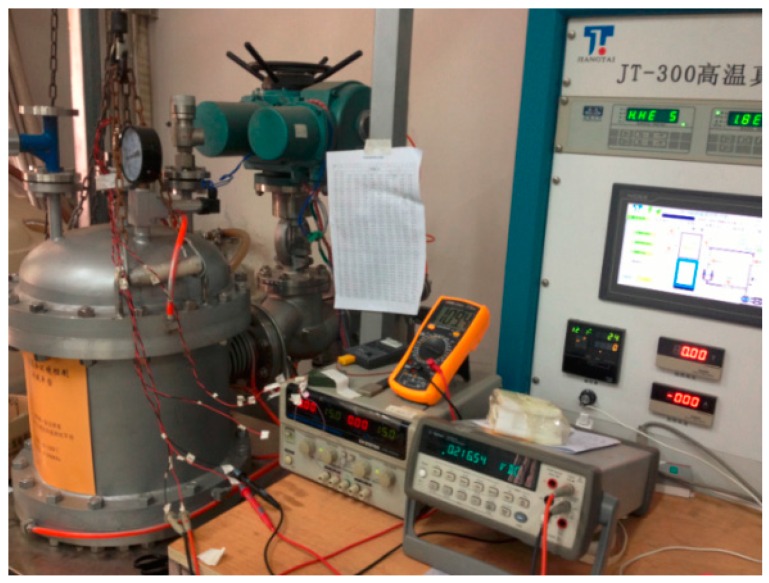
High-temperature and pressure calibration device developed by the authors.

The uncompensated high-temperature pressure sensor can be tested using the high-temperature and pressure calibration device shown in Figure 7. The sensor was calibrated in the ranges of 20–220 °C and 100–2000 kPa. 

**Figure 8:**
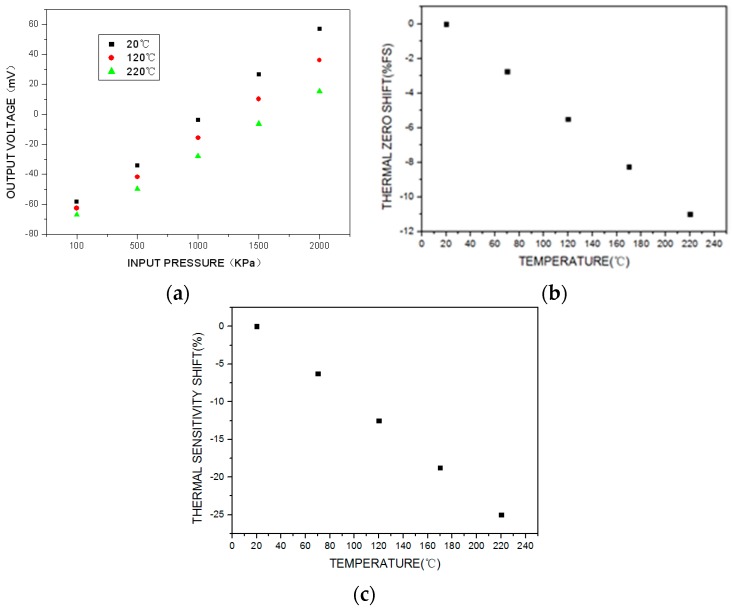
Test results for the uncompensated high-temperature pressure sensor: (**a**) output voltage calibration curve in the temperature and pressure environment; (**b**) thermal zero shift; (**c**) thermal sensitivity shift.

The test results are shown in Figure 8. Because of the parameter deviation among the piezoresistors of the MEMS pressure sensor and the residual stress caused by the fabrication process or a mismatch in the thermal expansion coefficients, the sensor’s initial offset voltage is negative and the output voltage decreases significantly as the temperature increases, as shown in Figure 8a. The thermal zero shift and thermal sensitivity shift are shown in Figure 8b,c. Over the working temperature range, the total accuracy is ±18%FS (full scale), the maximum thermal zero shift is −11%FS, and the maximum thermal sensitivity shift is −25%. The uncompensated high-temperature pressure sensor exhibits significant parameter drift over the whole working temperature range, which greatly affects the measurement accuracy.

**Figure 9:**
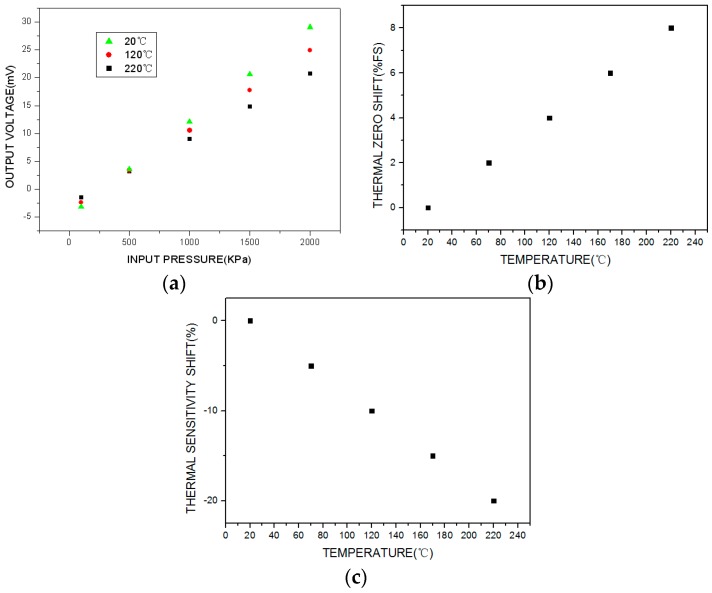
Test results for the compensated high-temperature pressure sensor with the traditional temperature compensation model and experiential arithmetic: (**a**) output voltage calibration curve in the temperature and pressure environment; (**b**) thermal zero shift; (**c**) thermal sensitivity shift.

The traditional temperature compensation model and experiential arithmetic shown in Figure 1 and Equations (1)–(3) were used to compensate for the output voltage temperature. The test results for the compensated sensor are shown in Figure 9. Over the working temperature range, it can be seen that the total accuracy is ±12%FS, the maximum thermal zero shift is +8%FS, and the maximum thermal sensitivity shift is −20%. Thus, traditional temperature compensation is relatively ineffective.

The passive resistor temperature compensation model and differential equations derived in this paper were applied to the bridge arm resistors at temperature thresholds of 20 °C and 220 °C and load pressures of 200 kPa and 600 kPa. The results are listed in Table 2. There are clearly large differences between the four initial bridge arm resistances, and some variation in resistance with load pressure and temperature coefficient due to process variables (such as photolithography) or the residual stress caused by the fabrication process.

**Figure 10:**
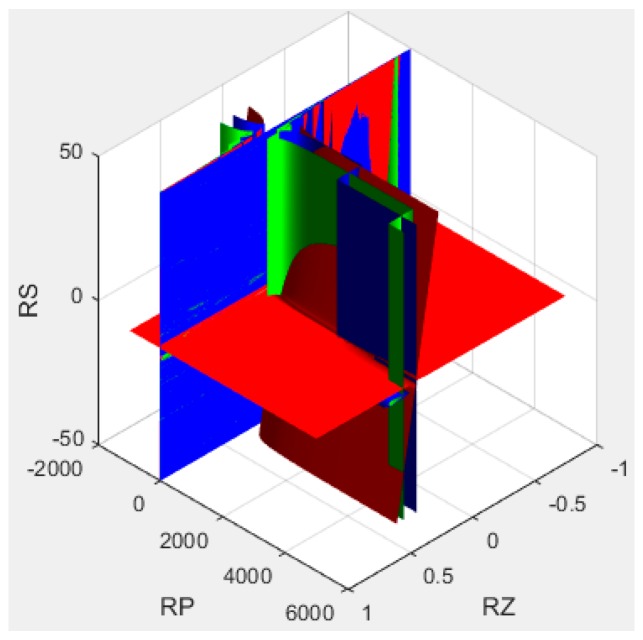
Solving equations by plotting the parameter space in MATLAB.

Because of the negative initial offset output voltage, the compensation model circuit shown in Figure 5a should be adopted. The algorithm for passive resistor temperature compensation shown in Equation (15) can be solved by examining the diagram in Figure 10, which was constructed using MATLAB from the test data in Table 2 (setting the offset output voltage U_0_ = 4 mV).

In the Figure 10, the red surface is drawn according to the first equation in Equation (15), representing the compensation of the offset voltage U_0_; the green surface is drawn according to the second equation in Equation (15), representing the compensation of the temperature coefficient of offset; the blue surface is drawn according to the third equation in Equation (15), representing the compensation of the temperature coefficient of sensitivity.

The parameter values were varied within the following compensation resistance ranges:

$$R_{Z}\in \left[0,\;200\;\Omega \right],R_{P}\in \left[1\;k\Omega ,1000\;k\Omega \right],R_{S}\in \left[1\;K\Omega ,30\;k\Omega \right]$$

From this, we obtained the minimal compensation resistance parameters:

$$R_{Z}=100\;\Omega ,R_{P}=180\;k\Omega ,R_{S}=22\;k\Omega$$

**Figure 11:**
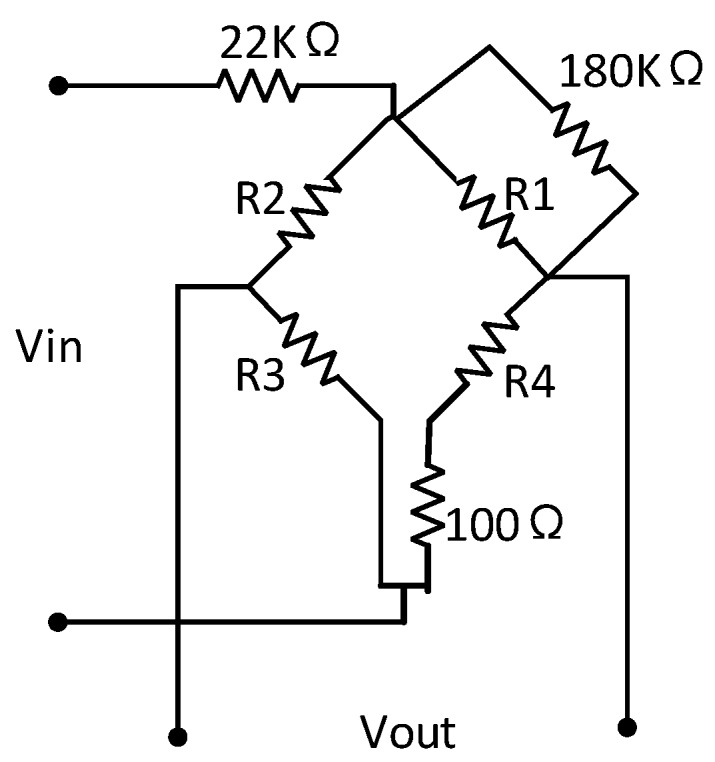
Passive resistor temperature compensation circuit using the data in Table 2.

The passive resistor temperature compensation circuit was then established using the data in Table 2. The resulting circuit is shown in Figure 11.

**Figure 12:**
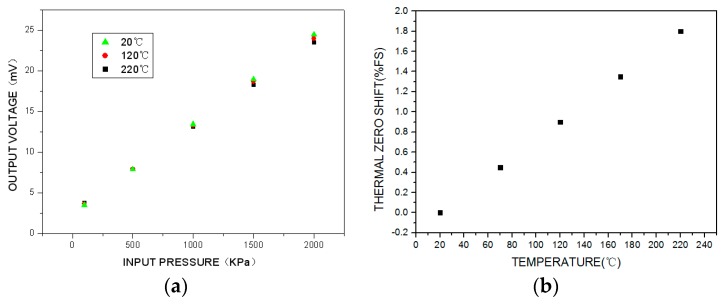

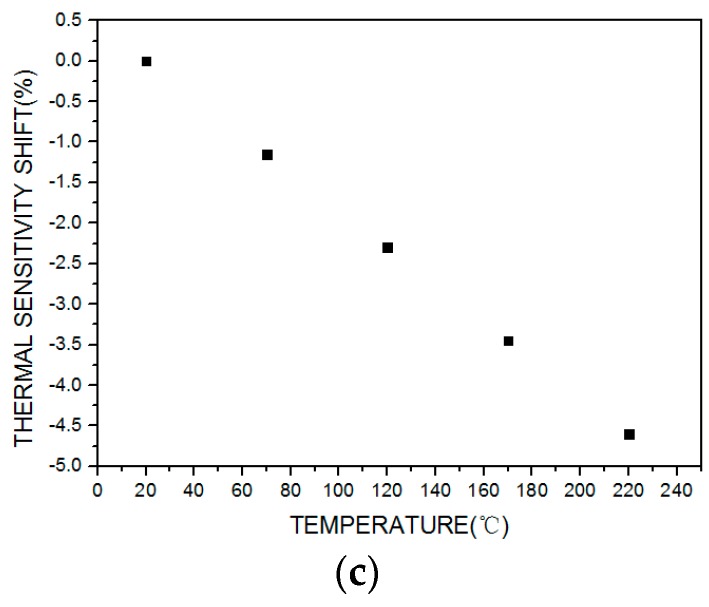
Test results for the compensated high-temperature pressure sensor with the passive resistor temperature compensation model and experiential arithmetic: (**a**) output voltage calibration curve in the temperature and pressure environment; (**b**) thermal zero shift; (**c**) thermal sensitivity shift.

The test results for the sensor under passive resistor temperature compensation are shown in Figure 12. Over the working temperature range, the total accuracy is ±1.5%FS, the maximum thermal zero shift is +1.8%FS, and the maximum thermal sensitivity shift is −4.6%.

From the above results, it is clear that the uncompensated sensor calibration curve (Figure 8) shows significant variation over the temperature range. The sensor calibration curve compensated by the traditional method has an improved total accuracy over the temperature range (Figure 9). However, the sensor calibration curve compensated by the proposed passive resistor temperature compensation (Figure 12) clearly exhibits the best accuracy over the whole temperature range. It is obvious that passive resistor temperature compensation is more effective than the traditional temperature compensation, as it resulted in higher measurement accuracy across the experimental temperature range. 

**Figure 13:**
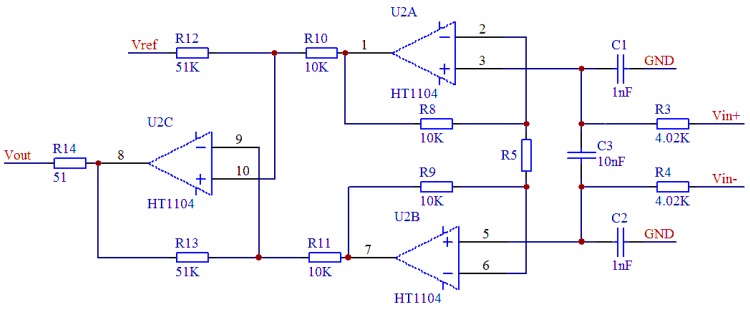
Schematic of a high-temperature signal-conditioning circuit.

One limitation of this compensation approach is its effect on the output sensitivity. As can be seen by comparing Figure 8 and Figure 12, the output sensitivity falls by 70% under the proposed method. To ameliorate this problem, we can use a high-temperature signal-conditioning circuit to improve the sensor’s output sensitivity, as shown in Figure 13. The main function of the circuit is to amplify the sensor output voltage signal from tens of millivolts to 0–5 V.

In Figure 13, V_in_+ and V_in_- are the output voltages of the bridge, V_ref_ is the output zero reference voltage of the circuit, and R5 (the gain adjustment resistor) determines the output voltage of the high-temperature signal-conditioning circuit, which can be expressed as:

(16) $$V_{\text{OUT}}=5.1\times \left(1+\frac{20K}{R5}\right)\times \left(V_{\text{in}+}-V_{\text{in}-}\right)+V_{\text{ref}}$$

**Figure 14:**
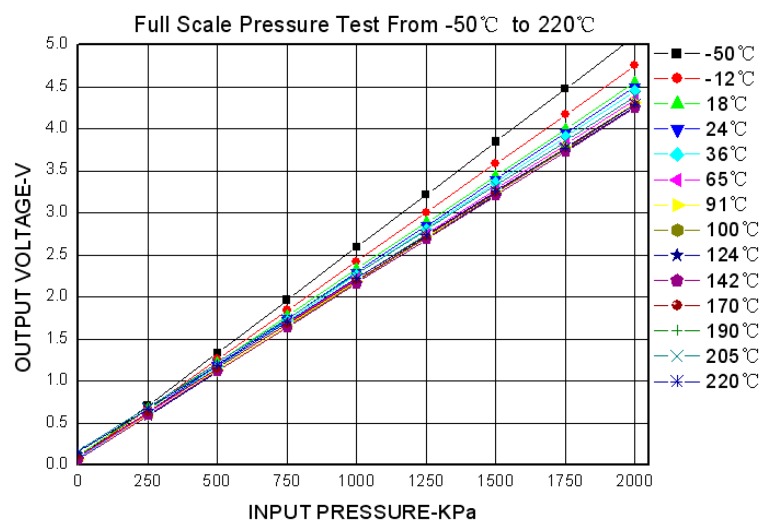
Pressure sensor calibration test results.

The test results for the high-temperature piezoresistive pressure sensor with passive resistor temperature compensation and the high-temperature signal-conditioning circuit are shown in Figure 14, and a performance comparison with the corresponding XTE-190 sensor (produced by KULITE) is listed in Table 3.

As can be seen from these results, the uncompensated high-temperature pressure sensor suffers from a significant degree of parameter drift across the whole working temperature range, but the sensor with passive resistor temperature compensation and the high-temperature signal-conditioning circuit can achieve similar levels of accuracy and temperature drift as the XTE-190. 

**Figure 15:**
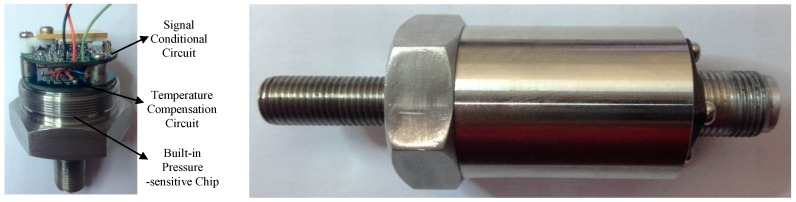
Sensor device pictures.

Additionally, in order to fully verify the effect of passive resistor temperature compensation, the same batch of six sensors has completed the temperature compensation calibration test by the same method, and the compensation effect reaches the same level. The sensor device pictures are shown in Figure 15.

## 5. Conclusions

In this paper, we have presented a broadly applicable method for passive resistor temperature compensation for high-temperature piezoresistive pressure sensors. The proposed method uses an algorithm based on the solution of differential equations. Our passive resistor temperature compensation technique is not affected by the characteristic deviation between bridge arm resistors or residual stress. Using only four bridge arm resistance measurements at different temperature thresholds and different load pressures, the temperature compensation circuit and compensation parameters can be determined computationally. Additionally, a high-temperature signal-conditioning circuit can be used to improve the output sensitivity of the compensated sensor. The compensation effect of the passive resistor temperature compensation on a high-temperature pressure sensor was demonstrated to be significantly better than that of the conventional compensation technique across a wide range of temperatures and pressures, suggesting that our method is worthy of popularization and application in sensor fabrication.

## Figures and Tables

**Table 1:** Measurement parameters for compensation model.

	R1	R2	R3	R4
(T0, P0)	R1(T0, P0)	R2(T0, P0)	R3(T0, P0)	R4(T0, P0)
(T0, P1)	R1(T0, P1)	R2(T0, P1)	R3(T0, P1)	R4(T0, P1)
(T1, P0)	R1(T1, P0)	R2(T1, P0)	R3(T1, P0)	R4(T1, P0)
(T1, P1)	R1(T1, P1)	R2(T1, P1)	R3(T1, P1)	R4(T1, P1)

**Table 2:** Test results for bridge arm resistors under different environmental conditions.

	R1 (kΩ)	R2 (kΩ)	R3 (kΩ)	R4 (kΩ)
(20 °C, 200 kPa)	4.54	4.533	4.82	4.71
(20 °C, 600 kPa)	4.52	4.588	4.8	4.743
(220 °C, 200 kPa)	6.8	6.728	7.2697	6.985
(220 °C, 600 kPa)	6.78	6.754	7.258	7.028

**Table 3:** Performance comparison of similar sensor parameters.

Parameters	Proposed Sensor	XTE-190
Operational mode	Absolute	Absolute
Pressure range	2 MPa	1.7 MPa
Compensated temperature range	+20–220 °C	+25–232 °C
Sensitivity (10 V power supply)	210 mV/100 kPa	8 mV/100 kPa
Thermal zero shift Thermal sensitivity shift	±1.2%FS/100F ±1.2%/100F	±1%FS/100F ±1%/100F
Total accuracy in compensation temperature range	±2%FS	±1.5%FS
